# Bioremediation of Heavy Metal-Contaminated Solution and Aged Refuse by Microbially Induced Calcium Carbonate Precipitation: Further Insights into *Sporosarcina pasteurii*

**DOI:** 10.3390/microorganisms13010064

**Published:** 2025-01-02

**Authors:** Dingxiang Zhuang, Weiheng Yao, Yan Guo, Zhengzheng Chen, Herong Gui, Yanyang Zhao

**Affiliations:** 1 National Engineering Research Center of Coal Mine Water Hazard Controlling, School of Resources and Civil Engineering, Suzhou University, Suzhou 234000, China; jiangwei3q@ahszu.edu.cn (W.Y.); guoyan@ahszu.edu.cn (Y.G.); chenzhzh@ahszu.edu.cn (Z.C.); guiherong@ahszu.edu.cn (H.G.); 2 Shandong Provincial Key Laboratory of Depositional Mineralization and Sedimentary Minerals, College of Earth Science and Engineering, Shandong University of Science and Technology, Qingdao 266590, China; zhaoyanyang@sdust.edu.cn; 3 Key Laboratory of Deep Oil and Gas, China University of Petroleum, Qingdao 266580, China

**Keywords:** contaminated solution, aged refuse, bioremediation, *Sporosarcina pasteurii*, MICP

## Abstract

Recently, the ability of microbial-induced calcium carbonate precipitation (MICP) to remediate heavy metals has been widely explored. *Sporosarcina pasteurii* was selected to remediate heavy metal-contaminated solution and aged refuse, exploring the feasibility of *Sporosarcina pasteurii* bioremediation of heavy metals and analyzing the changes in heavy metal forms before and after bioremediation, as well as the mechanism of remediation. The results showed that *Sporosarcina pasteurii* achieved remediation rates of 95%, 84%, 97%, and 98% for Cd, Pb, Zn, and Cr (III) in contaminated solution, respectively. It also achieved remediation rates of 74%, 84%, and 62% for exchangeable Cd, Pb, and Zn in aged refuse, respectively. The content of exchangeable Cr (III) before bioremediation was almost zero. The content of heavy metals with exchangeable form and carbonate-bounded form in aged refuse decreased after bioremediation, while the content of heavy metals with iron–manganese oxide binding form and residual form increased. Simultaneously, the presence of Fe and Al components in aged refuse, as well as the precipitation of calcium carbonate produced during the MICP process, jointly promotes the transformation of heavy metals into more stable forms.

## 1. Introduction

A large number of informal landfills are operating beyond their capacity, and this has given rise to a series of problems that need to be solved. In the process of sanitary landfill, the leachate with high metal content formed during rainfall, the water produced by the garbage itself after passing through the garbage layer, and the soil cover layer pollute the surrounding surface water and groundwater [1,2]. At the same time, the mining and expansion of old landfills have also given rise to the problem of disposal of aged refuse. The aged refuse obtained from mining and screening has a high organic matter content, is rich in trace elements and microbial flora, and has the potential for resource utilization as soil for landscaping and fruit forests [3,4]. However, established studies have shown that mined and screened aged refuse tend to contain high concentrations of heavy metals. For example, Wu et al. (2022) found that Pb, Cr, and Cd exceeded the standards in a landfill [5]. Alam et al. (2020) showed that the Zn content in a landfill was 250% higher than the background value of natural soil, and Jimenez et al. (2023) found that industrial wastes such as leather contained a large amount of Cr(III), which caused serious pollution after landfilling [6,7]. Therefore, to prevent landfills from polluting the surrounding environment and in the process of resource utilization, scientific and reasonable remediation means are adopted for stabilization [8,9,10].

Common soil heavy metal remediation technologies include physical remediation technologies (isolation encapsulation, electrothermal remediation, and electrokinetic remediation), chemical remediation technologies (chemical amelioration remediation and chemical drenching remediation), and bioremediation technologies (phytoremediation and microbial remediation) [11,12]. The principles include isolation encapsulation, leaching removal, and solidification stabilization. Aged refuse needs to be treated off-site for resource use, so physical treatment techniques such as segregated encapsulation and electro-remediation are not applicable. Electrothermal remediation method, chemical improvement remediation method, and chemical washing remediation method are serious modification of aged refuse, destroying the soil structure and reducing the fertility of the soil, which are not conducive to the further resource utilization of aged refuse [13,14,15]. Phytoremediation techniques are inefficient, and heavy metals are easily transported during the remediation process [16]. Therefore, bioremediation technologies with features such as green energy and high efficiency have begun to emerge in research.

Recently, the application potential of microbial-induced carbonate precipitation (MICP) technology in the field of heavy metal remediation has been widely explored [17,18,19]. The mechanisms of MICP include biological mechanism and precipitation mechanism. Almost all organisms in nature are mineralized in some way, such as the bones and teeth of mammals, the shells of the mollusk abalone, and the shells of arthropod barnacles [20]. A variety of mineralization models exists in microorganisms alone, including nitrate reduction models, sulfate models, denitrification models, and so on. The carbonate precipitations model through urease microbial hydrolysis of urea is the most direct and controllable form of the MICP, which can produce large amounts of carbonate in a short period of time [21]. Therefore, urease microbial hydrolysis of urea is widely used in a microbial mineralization model. Urease-producing microorganisms represented by *Sporosarcina pasteurii* secrete urease through metabolism and hydrolyze urea in the environment. As an organic small molecule, urea can enter the bacterial cells via free diffusion, and it will pour into the bacteria in large quantities when the concentration of urea in the environment is high. The hydrolysis of urea is catalyzed by urease inside the bacteria, and the hydrolysis process can be roughly divided into two steps. First, urea is hydrolyzed to carbamate and ammonia molecules catalyzed by urease. Then, the carbamate is spontaneously hydrolyzed to form carbonic acid and ammonia molecules. Ultimately, the products of urea hydrolysis are ammonia molecules and carbonic acid. At physiological pH, the carbonic acid is protonated, and the ammonia molecules are protonated by water molecules, resulting in an increase in pH value. The whole reaction process is shown in the following equations.

(1) $$CO{\left(NH_{2}\right)}_{2}+H_{2}\to NH_{3}+NH_{2}COOH$$

(2) $$NH_{2}COOH+H_{2}O\to H_{2}CO_{3}+NH_{3}$$

(3) $$H_{2}CO_{3}\to 2H^{+}+CO_{3}^{2-}$$

(4) $$NH_{3}+H_{2}O↔NH_{4}^{+}+OH^{-}$$

Calcium carbonate precipitation is a fairly simple chemical process controlled by four key factors: calcium concentration, the concentration of soluble inorganic carbon, pH value, and the presence of nucleation sites [22]. Calcium carbonate precipitation requires sufficient concentrations of calcium and carbonate ions so that the product of the ionic activities (*α*) of the two ions exceeds the dissolution equilibrium constant (*Kso*) of calcium carbonate, as shown in Equations (5) and (6). The ratio of the ionic activity product to the dissolution equilibrium constant defines the degree of saturation of the system as *Ω*. When *Ω* > 1, this indicates that the solution system is supersaturated and may precipitate calcium carbonates. Generally, under natural conditions, the precipitation process described above will be very slow, but microorganisms can control or induce calcium carbonate precipitation by altering any of the above key factors affecting calcium carbonate precipitation. This results in the production of large amounts of calcium carbonate in a short period of time.

(5) $$Ca^{2+}+CO_{3}^{2-}\to CaCO_{3}$$

(6) $$\Omega =\alpha (Ca^{2+})\alpha (CO_{3}^{2-})/K_{SO}(K_{SO25\;{}^{\circ}C}=4.8\times 10^{-9})$$

Microorganisms are utilized to immobilize heavy metal ions and reduce the mobility, activity, and biotoxicity of heavy metals, which have the advantages of short treatment cycle, good remediation effect, small soil disturbance, and economic and environmental protection. Some studies have shown that heavy metal-tolerant microorganisms isolated from polluted environments, including *Sporosarcina pasteurii*, stand out due to their environmental adaptability and high urease expression [23,24]. Some scholars have investigated the potential of *Sporosarcina pasteurii* for the remediation of heavy metals; for example, Pagnucco et al. (2023) found that the removal of heavy metals could be increase up to 88% when the concentration of Zn^2+^, Cd^2+^, and Pb^2+^ was 2000 mg/L [25]. Other researchers similarly demonstrated that *Sporosarcina pasteurii* could effectively remediate Cd^2+^ and Pb^2+^, but Khan et al. (2022) found that the bioremediation effect decreased rapidly when Zn^2+^ concentration was higher than 113 mg/L [26]. In addition, Yang et al. (2024) found that *Sporosarcina pasteurii* could effectively reduce the effective state of Pb in the soil [27]. Overall, established studies on MICP remediation of heavy metals have shown that *Sporosarcina pasteurii* can be effective in removing Cd, Pb, and Zn from contaminated solutions or contaminated soils within a certain range [28]. However, the pollution genesis, chemical composition, and microflora components of the aged refuse are significantly different from those of the contaminated soils in the established studies. Aged refuse contains a large number of clay and powder particles, with small porosity, and its chemical composition is complex, containing a large amount of organic matter. Therefore, in low porosity, low oxygen, and high organic matter environments, microorganisms are inactivated, or the soil particles prevent the reaction from proceeding further, all of which can have a significant impact on the biomineralization process [29]. Therefore, the conclusions of MICP remediation of contaminated solutions or common contaminated soils may not be applicable for the bioremediation of aged refuse, and it is necessary to carry out a systematic feasibility study of bioremediation for aged refuse contaminated with different heavy metals [30].

In this work, the experimental study of microbial remediation of contaminated solution and aged refuse was carried out at different concentrations using *Sporosarcina pasteurii* as the test strain. Aged refuse is formed in landfills after many years of landfilling. Specifically, aged refuse refers to refuse that has been landfilled in a landfill for many years and has essentially stabilized to the point where it is ready to be mined for use. Aged refuse contains four major components: minerals, organic matter (including living organisms), water, and gasses. Minerals are its main solid component, forming the skeleton and determining the hydrodynamic properties. Organic matter improves the substrate structure and provides a nutritious environment for microorganisms. The feasibility of bioremediation of heavy metals by *Sporosarcina pasteurii* was investigated; the transformation of heavy metals in the aged refuse before and after bioremediation was analyzed, and the microscopic morphology and the chemical composition of the aged refuse before and after the bioremediation were determined with the help of SEM and XRD to investigate its mechanism.

## 2. Materials and Methods

### 2.1. Source and Characterization of Aged Refuse

Contaminated aged refuse was taken from a landfill in Suzhou, Anhui Province, China (116°~119° N; 33°~39° E), as shown in Figure S1. The main steps in obtaining aged refuse from the landfill included excavation, sorting, and screening. Excavators were first used to excavate the refuse in the landfill, and the excavated refuse was then transported to the refuse treatment plant using enclosed waste haulers. Through a series of refuse sorting equipment (conveyor, tumbler screen, wind separator, and magnetic separator), recyclable materials, combustible materials, and organic materials were sorted out from the refuse. The screened refuse was sent to a tumbler screen via a conveyor to be screened, and materials less than 50 mm were categorized as contaminated aged refuse.

**Figure 1:**
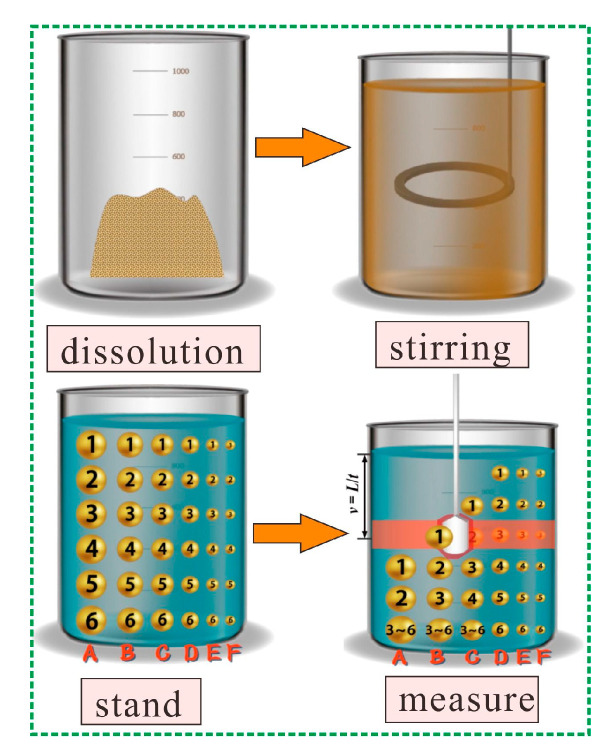
The experimental procedure for the densitometric method.

The size distribution of the contaminated aged refuse was analyzed using the densitometric method. The contaminated aged refuse and distilled water were added in turn in a 1000 mL measuring cylinder. The solution was homogenize from top to bottom after a thorough mixing. That was to say that particles of different sizes were evenly distributed in the solution, with the largest particles being numbered A, and the smallest particle sizes being numbered F. The content of particle size A to F in each layer of the solution was exactly the same. After stopping the stirring, the large particles will sink faster, and the small particles will sink slower. If a densitometer was inserted at this time, it would stay at a certain position in the solution. The rate of sinking of particle size B, which represented the particle size of the aged refuse, was determined using a scale and the fall time of the densitometer [31,32]. The experimental procedure for the densitometric method is shown in Figure 1.

### 2.2. The Measurement of Heavy Metal Content

The chemical indicators such as pH value, heavy metal content, organic matter, and cation exchange capacity were measured. Among them, the heavy metal content of the aged refuse was extracted via a graphite abatement method and then measured using an atomic absorption photometer (TAS-986, Beijing Purkinje General, Beijing, China). Graphite ablation converts the heavy metal elements in the sample into measurable ionic forms via the action of heat and acid. This method is effective in destroying the organic material in the sample and releasing the heavy metal ions [33]. Atomic absorption spectrophotometer utilizes the principle of atomic absorption spectroscopy to quantitatively analyze the heavy metal content of a sample by measuring how many gaseous atoms of a specific element absorb a light beam [34]. The samples were placed in a tetrafluoroethylene ablation tank, and 5 mL of nitric acid was added, and then placed in a microwave ablator for ablation. Upon completion, the solution was placed on a hotplate, heated to evaporate the NO vapor, and reduced to 2–3 mL, followed by the addition of 1% HNO_3_ to a volume of 25 mL and shaking well. Standard solutions of each element were aspirated and sequentially diluted to different concentration gradients with 1% HNO_3_ solution. The absorption values were then determined according to the specific conditions.

Appropriate amounts of Cd, Pd, Zn and Cr (III) standards were weighed, transferred to a volumetric flask, and dissolved by adding distilled water with thorough stirring. Distilled water was continuously added to the volumetric flask until the concentration was 1000 ug/mL. Different volumes of the solution were pipetted into the volumetric flask each time to obtain different concentrations of the standard solutions. The standard curve was plotted according to the concentration and absorbance [35].

### 2.3. Experimental Strain

The strain used for the test was *Sporosarcina pasteurii*, and the lyophilized powder of the bacterium was purchased from the China General Microbial Strain Preservation and Management Center (CGMCC) under the strain number CGMCC 1.3687. The bacterial broth was cultured using a culture medium consisting of 20 g/L yeast extract, 10 g/L NH_4_Cl, 10 mg/L MnSO_4_·H_2_O, and 24 mg/L NiCl_2_·6H_2_O. The above components were mixed proportionally with deionized water, and the pH value of the culture solution was adjusted to 8.5 with 1 mol/L NaOH. Then, the culture solution was autoclaved at 121 °C for 30 min, and the bacterial liquid was inoculated with the culture solution at a ratio of 1:100 by volume on an aseptic operating table. After inoculation, the culture solution was incubated at a constant temperature on a shaker (30 °C, 170 r/min) for 24 h. The values of OD_600_ were obtained using an ultraviolet spectrophotometer during microbial cultivation to obtain the different bacterial concentrations. In addition, the urease activity of bacteria was determined using an electrical conductivity meter (SIN-TDS210-C, Beijing Purkinje General, Beijing, China). The conductivity method for determining bacterial urease activity is based on the principle of reflecting urease activity by measuring the change in conductivity in the reaction solution. Specifically, urease catalyzes the reaction between urea and water to produce urea. Further hydrolysis of urea produces ammonia and carbon dioxide, leading to an increase in ion concentration in the reaction solution, which affects the conductivity. By recording the change in conductivity during the reaction, the urease activity can be indirectly determined. The steps and precautions for the measurement can be referred to in Li et al. (2021) and Zhang et al. (2014) [36,37].

### 2.4. Bioremediation of Contaminated Solution

Due to the extremely complex composition of the waste leachate, which contains a large number of organic pollutants, it is difficult to control the pollution conditions. Therefore, bioremediation of the contaminated solution and aged refuse was carried out using Cd-, Pb-, Zn-, and Cr(III)-contaminated solution and aged refuse as the remediation targets, respectively. The former explored the feasibility of bioremediation of contaminated solution by *Sporosarcina pasteurii*, while the latter explored the feasibility of bioremediation of aged refuse, the effect of bioremediation on the morphology of heavy metals, and the mechanism of bioremediation. The specific experimental program was as follows: Test 1 was the bioremediation of pollution solutions. Four test groups of Cd, Pb, Zn, and Cr (III) were set up, and at the same time, each test group contained three concentration gradients. The numbers of the test groups were A_1_, A_2_ and A_3_, and the values of A_2_ and A_3_ are 10 times and 100 times the concentration value of A_1_, respectively. The details are shown in Table 1.

#### 2.4.1. Preparations Before the Test 1

CdCl_2_, PbCl_2_, ZnCl_2_, and CrCl_3_ were added to deionized water to prepare a high concentration of heavy metal reserve solution. A urea–calcium chloride nutrient solution with a concentration of 0.5 mol/L was prepared. The test was carried out in 50 mL centrifuge tubes, which were numbered and weighed for mass, recorded as *m*_0_.

#### 2.4.2. Process of the Test 1

The bacterial solution, the nutrient solution, and the heavy metal reserve solution were mixed with a ratio of 2:1:1 by volume, and the total volume of the solution after mixing was 40 mL. The concentration of each component is shown in Table 1. The bioremediation was completed by shaking for 24 h at room temperature and at 220 r/min.

#### 2.4.3. Determination of Test 1 Parameters

Subsequently, the supernatant and precipitate were obtained via centrifugation (3000 r/min, 10 min). The supernatant was filtered and then diluted, and the heavy metal ion concentrations were determined before and after bioremediation using an atomic absorption spectrophotometer.

The centrifuge tubes with precipitates were placed in an oven (60 °C, 24 h), and the dried mass was recorded as *m*_24_. Bioremediation rates *η* were calculated as shown in Equation (7). In addition, the mass increase *δ* was obtained as shown in Equation (8). Here, *i*_0_ and *i*_24_ are the ion concentrations at different bioremediation time of 0 and 24 h, respectively; *m*_0_ and *m*_24_ are the precipitation–centrifuge tube masses at different bioremediation time of 0 and 24 h, respectively [38].

(7) $$\eta =\frac{i_{0}-i_{24}}{i_{0}}\times 100\%$$

(8) $$\delta =m_{24}-m_{0}$$

### 2.5. Bioremediation of Contaminated Aged Refuse

Test 2 was a bioremediation of contaminated aged refuse. The heavy metal species were the same as in Test 1, and three concentration gradients were set up, named B_1_, B_2_, and B_3_. The values of B_2_ and B_3_ are 10 times and 100 times the concentration value of B_1_, respectively. The concentration of each component is shown in Table 2.

#### 2.5.1. Preparations Before the Test 2

The contaminated aged refuse was prepared by mixing a high concentration of heavy metal reserve solution (same as in Section 2.4.1) and aged refuse according to a 20:1 ratio (mL/g). Then, the mixed solution was shaken (220 r/min, room temperature, 24 h), centrifuged (3000 r/min, 10 min), and dried (60 °C, 24 h) to obtain the contaminated aged refuse. The content of heavy metals in different forms was determined using the Tessier five-step extraction method. The steps and precautions for the measurement can be referred to in Burachevskaya et al. (2022) [39].

#### 2.5.2. Process of the Test 2

The bioremediation of aged refuse was carried out in a 50 mL centrifuge tube by mixing the bacterial solution, nutrient solution, and aged refuse in a ratio of 10:10:1 (mL:mL:g), with a total volume of the solution after mixing of 40 mL. The concentrations of each component are shown in Table 2. The bioremediation was completed by shaking for 24 h at room temperature and at 220 r/min.

#### 2.5.3. Determination of Test 2 Parameters

The content of heavy metals in different forms was determined using the Tessier five-step extraction method. Bioremediation rates *η* and mass increase *δ* were obtained as shown in Equations (7) and (8).

### 2.6. X-Ray Diffraction Analysis

The precipitates in the contaminated solution and the aged refuse before and after bioremediation were analyzed using X-ray diffraction (XRD, Bruker D8 Advance, Ettlingen, Germany). Specifically, 20 g of the sample was removed and dried in an oven at 50 °C for 4 h. Subsequently, it was sieved utilizing a 200-mesh sieve to ensure uniform size was obtained. The experimental parameters were set so that Cu’s K*α* radiation was used with scanning angles (2*θ*) from 10° to 80° and scanning speeds of 2° min^−1^ (with a 0.02° step size). The diffraction pattern information was collected after the test was completed. Data processing was carried out using a specialized Jade software 9.0, which included curve smoothing, background deduction, peak searching, and phase identifying to obtain information about the mineral composition and crystal structure of the sample [40].

### 2.7. Scanning Electron Microscopy and Energy Dispersive Spectroscopy

To further analyze the microstructure and element distribution of the contaminated aged refuse before and after bioremediation, SEM combined with EDS (HITACHI, S-4800, Tokyo, Japan) as used for observation. Due to the lack of conductivity of the sample, it is necessary to perform a coating and conductivity treatment on the sample before conducting the experiment. The processed sample was fixed on the stage with a conductive adhesive, and the distance between the probe and the sample was adjusted; the best images after multiple scanning with the scanner was vacuumized. The operating electron accelerating voltage was from 300 V to 30 kV for high-resolution secondary electron imaging and elemental analysis [41].

## 3. Results

### 3.1. The Size Distribution of the Contaminated Aged Refuse

**Figure 2:**
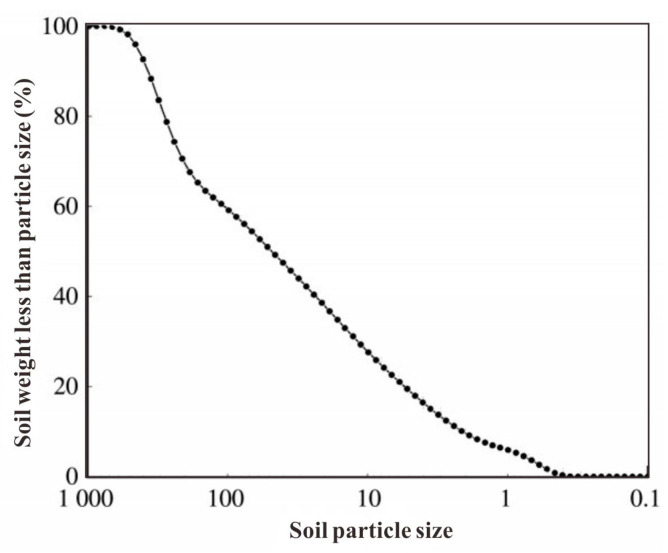
The size distribution of the contaminated aged refuse.

The size distribution of the contaminated aged refuse analyzed using the densitometric method is shown in Figure 2. The results showed that the content of clay particles in aged refuse (particle size less than 0.005 mm) was 17.96%; the content of powder particles (particle size 0. 005–0.075 mm) was 36.46%, and the content of sand particles (particle size 0.075–2 mm) was 54.42%. This illustrated the fact that the content of sand particles in the contaminated aged refuse was the highest, but the content of clay particles was the lowest.

### 3.2. Basic Chemical Characteristic Parameters of Contaminated Aged Refuse

The results of pH value, heavy metal content, organic matter, and cation exchange capacity are shown in Table 3. It can be seen that the pH value of contaminated aged refuse was 8.4. Comparing the heavy metal content of contaminated aged refuse with the third level standard value of “Greening Planting Soil”, the Zn and Cr contents were 1230 mg/kg and 256 mg/kg, respectively, which exceeded the standard value [42]. The results also showed that the organic matter and cation exchange were higher than the technically required values of soil fertility for green planting, and it has the potential for resource utilization.

### 3.3. Growth and Characteristics of Bacteria

**Figure 3:**
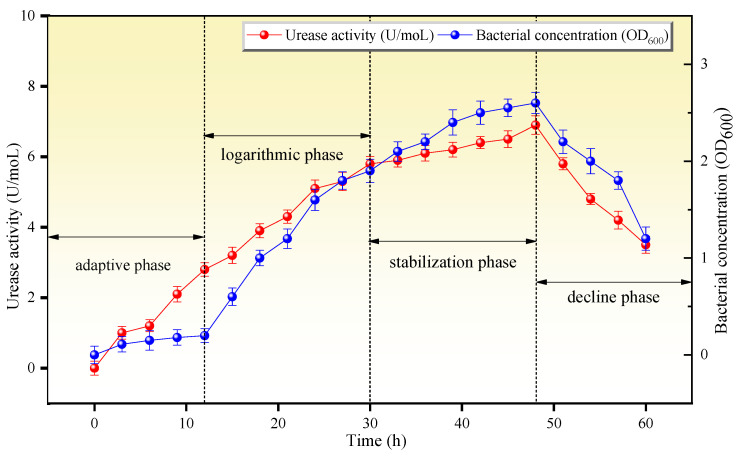
Microbial growth process curves.

The liquid culture medium was made of 20 g/L yeast extract, 10 g/L NH_4_Cl, 10 mg/L MnSO_4_·H_2_O, and 24 mg/L NiCl_2_·6H_2_O. Bacterial optical density and conductivity for different cultivation durations were measured to obtain the bacterial solution concentration (OD_600_) and urease activity at every 4 h time point. As shown in Figure 3, bacterial growth roughly went through four stages: the adaptive phase, the logarithmic phase, the stabilization phase, and the decline phase. Bacterial growth entered a relatively stable phase after 24 h of cultivation, and the number of microorganisms and urease activity reached a peak value at 48 h, corresponding to an OD_600_ value of 2.6 and a urease activity of 6.9 mmol/min. Therefore, *Sporosarcina pasteurii* cultured for 48 h was selected for the experiment, and both the bacterial solution concentration and urease activity reached their highest values, which was beneficial for inducing the precipitation of minerals.

### 3.4. Analysis of Bioremediation of Contaminated Solution

**Figure 4:**
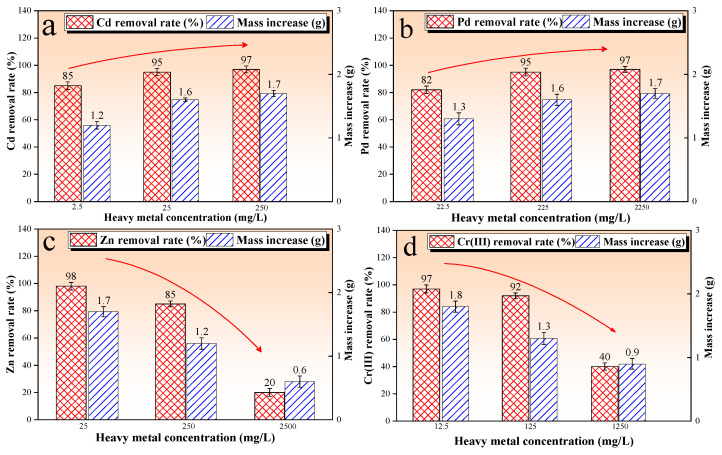
Bioremediation rates and quality increase in the test of bioremediation of contaminated solution; (**a**) the Cd-contaminated solution; (**b**) the Pd-contaminated solution; (**c**) the Zn-contaminated solution; (**d**) the Cr(III)-contaminated solution.

Figure 4 shows the results of the bioremediation of the contaminated solution test, that is, the bioremediation rate and mass increase were plotted against the heavy metal species and concentrations. The bioremediation rate directly reflected the bioremediation effect. In addition, CO_3_^2−^ produced via the hydrolysis of urea during the MICP process forms carbonate precipitates with Ca^2+^ and other metal ions. Therefore, the mass increase can indirectly characterize the microbial activity. The results showed that the bioremediation rates of the Cd and Pb test groups were higher than 85% and 82%, respectively, and the mass gain was around 1.7 g (as shown in Figure 4a,b). This indicated that *Sporosarcina pasteurii* consistently maintained high activity and efficiently remediated Cd^2+^ and Pb^2+^ in the concentration range studied. The bioremediation rate reached 98% when the initial concentration of Zn^2+^ was low (25–250 mg/L). However, the bioremediation rate plummeted to 5% when the concentration was increased to 2500 mg/L. In addition, the mass increase showed the same trend, decreasing from 1.7 g to 0.6 g (as seen in Figure 4c). The results also indicate that *Sporosarcina pasteurii* can effectively remediate Zn^2+^ but only in the low concentration range. In addition, the bioremediation rate of the Cr(III) test group reached up to 97%, and decreased to 40% as the Cr(III) concentration increased to 1250 mg/L. The corresponding mass increase showed a decreasing trend from 1.8 g to 0.9 g with the increase in the initial Cr(III) concentration (Figure 4d). Overall, *Sporosarcina pasteurii* has the ability to remediate Cd^2+^-, Pb^2+^-, Zn^2+^-, and Cr(III)-contaminated solutions.

### 3.5. Analysis of Bioremediation of Contaminated Aged Refuse

**Figure 5:**
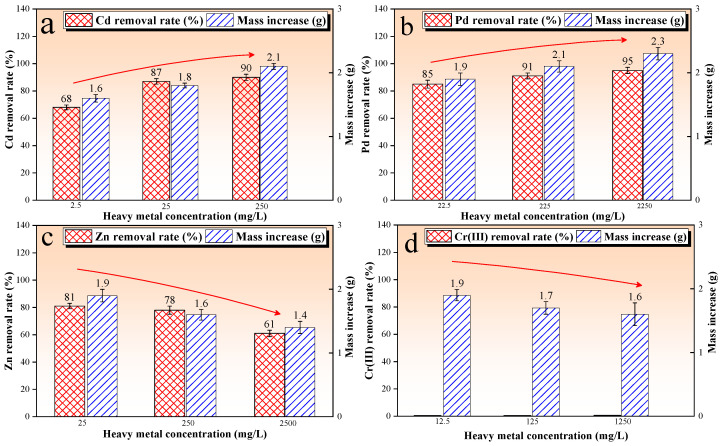
Bioremediation rates and quality increase in the test of bioremediation of contaminated aged refuse; (**a**) the Cd-contaminated aged refuse; (**b**) the Pd-contaminated aged refuse; (**c**) Zn-contaminated aged refuse; (**d**) the Cr(III)-contaminated aged refuse.

The results of the bioremediation of the contaminated aged refuse tests are shown in Figure 5. Figure 5a,b show that *Sporosarcina pasteurii* can efficiently and stably remediate exchangeable Cd^2+^ and Pb^2+^ in contaminated aged refuse. The bioremediation rate was higher than 68% and 85%, respectively. The mass increased up to 2.3 g. With the increase in the initial concentration of Zn^2+^, the bioremediation rate decreased from 81% to 61% (as shown in Figure 5c). The bioremediation rate for the Cr(III) test group was zero because the exchangeable state Cr(III) content was almost zero (as seen in Figure 5d). In addition, the mass increase was consistently between 1.4 g and 1.9 g for each test group (Figure 5a–d). Overall, *Sporosarcina pasteurii* can effectively remediate Cd-, Pb-, and Zn-contaminated aged refuse.

### 3.6. Analysis of Heavy Metals Forms of Contaminated Aged Refuse

**Figure 6:**
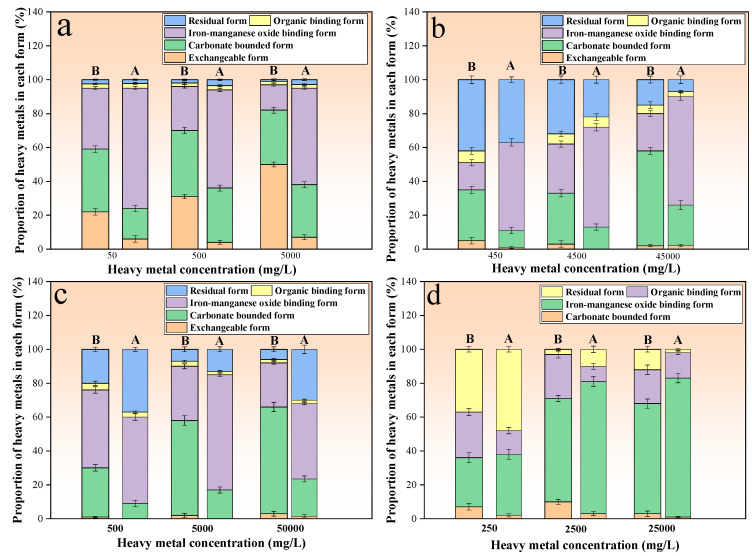
Percentage of various forms of heavy metals of contaminated aged refuse before and after bioremediation; (**a**) the Cd-contaminated aged refuse; (**b**) the Pd-contaminated aged refuse; (**c**) the Zn-contaminated aged refuse; (**d**) the Cr(III)-contaminated aged refuse; B, before bioremediation; A, after bioremediation.

Heavy metals exist in contaminated aged refuse and have different forms, including residual form, organic binding form, iron–manganese oxide binding form, carbonate-bound form, and exchangeable form, among which carbonate-bound and exchangeable heavy metals have stronger mobility and activity, and are the main targets for bioremediation [43,44]. Figure 6 compares the changes in the proportion of heavy metals in contaminated aged refuse before and after bioremediation. The results showed that there were some differences in the proportion of heavy metals in each form when the heavy metal species and concentrations were different before bioremediation, but they were mainly in the exchangeable form, carbonate-bound form, and iron–manganese oxide binding form. Specifically, the exchangeable form, carbonate-bound form, and iron–manganese oxide binding form of Cd-contaminated aged refuse were more prevalent before bioremediation, as shown in Figure 6a. The carbonate-bound form and iron–manganese oxide binding form of Pd-contaminated and Zn-contaminated aged refuse were more prevalent before bioremediation, as shown in Figure 6b,c. Figure 6d shows that the iron–manganese oxide binding form of Cr(III)-contaminated aged refuse accounted for a higher proportion than that of the other heavy metals forms.

After bioremediation, the heavy metal contents in the exchangeable form and carbonate-bound form decreased, and the heavy metal contents in the iron–manganese oxide binding form and residual form increased; the heavy metal contents in the organic binding form remained almost unchanged. Although there were some differences in the proportion of heavy metals in various forms when the heavy metal species and concentrations were different, the carbonate-bound form and iron–manganese oxide binding form were mainly dominant. Specifically, Figure 6a shows that the content of the exchangeable form and carbonate-bound form of Cd-contaminated aged refuse decreased from 26–47% and 31–34% to 4–6% and 17–30%, respectively, and the content of iron–manganese oxide binding form increased from 17–34% to 58–70%. The content of the carbonate-bound form of Pb-contaminated aged refuse decreased from 29–55% to 10–26%, and the iron–manganese oxide binding form increased from 18–29% to 54–67% (Figure 6b). The content of carbonate-bound form of Zn-contaminated aged refuse decreased from 28–62% to 10–19%, and the content of iron–manganese oxide binding form increased from 25–45% to 47–66% (Figure 6c). In addition, the iron–manganese oxide binding form of Cr(III)-contaminated aged refuse increased from 30–65% to 32–81% (Figure 6d). In summary, Cd, Pb, Zn, and Cr (III) in the contaminated aged refuse were converted to more stable forms after bioremediation treatment, and the mobility and activity of the heavy metals were effectively reduced.

### 3.7. Mineral Compositions of Contaminated Solution and Aged Refuse

**Figure 7:**
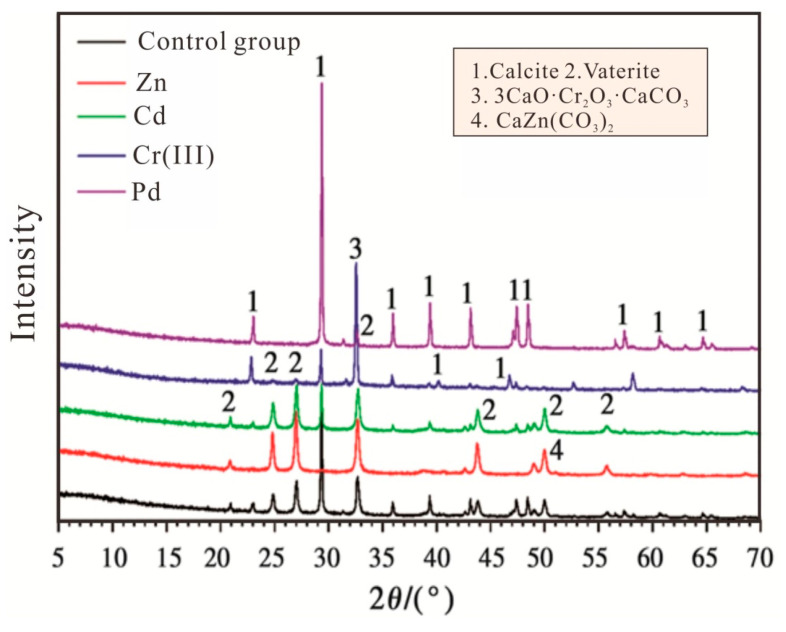
XRD results of the contaminated solution.

Figure 7 shows the XRD results of the bioremediation of the contaminated solution test. Solid phase precipitations in the control group were dominated by calcite and vaterite and marked with the numbers 1 and 2. However, the main component of the solid phase precipitation of the Cr(III) test group was calcium carbonate chromium (3CaO·Cr_2_O_3_·CaCO_3_) and was marked with the number 3. In contrast to Cr_2_O_3_ generated using pure chemical equilibrium simulations, Cr(III) formed precipitates in the form of CrO_4_^2−^ in the presence of microorganisms, and the remediated products were more complex. In addition, the precipitates in the Zn test group contained a small amount of CaZn(CO_3_)_2_ and was marked with the number 4. The results of the Cr(III) and Zn test groups indicate that *Sporosarcina pasteurii* can remove heavy metal ions by biomineralization, that is, the formation of oxides or carbonate precipitation. However, no corresponding oxide and carbonate precipitations were found in the solid phase precipitations of the Cd and Pb test groups due to the main components comprising calcite and vaterite, which was the same as the control group.

**Figure 8:**
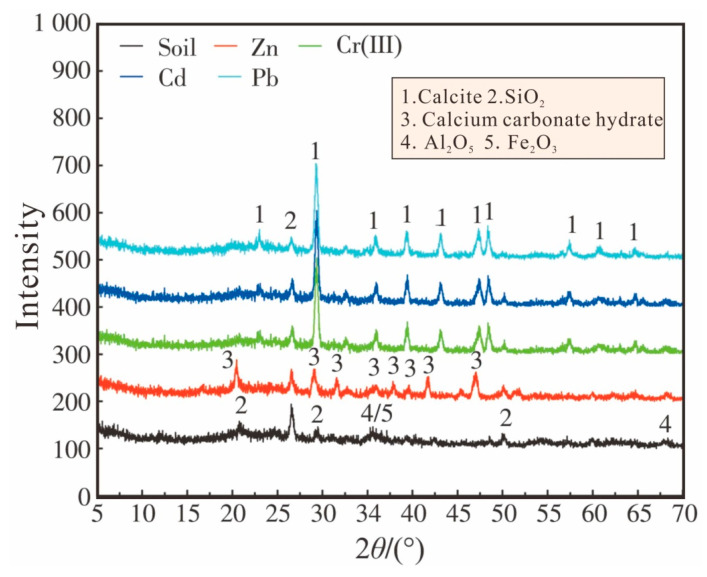
XRD results of contaminated aged refuse.

The XRD analyses of the contaminated aged refuse are shown in Figure 8. The results show that the main components of the soil before bioremediation were SiO_2_, Al_2_O_5_, and Fe_2_O_3_ and were marked with the numbers 2, 4, and 5, indicating the presence of iron oxide and aluminosilicate mineral. The precipitate in the Zn test group contained calcium carbonate hydrate marked with the number 3. In addition, calcites marked with the number 1 were found in Cd, Pb, and Cr(III) test groups. In addition, no other carbonate crystals were detected via XRD. Therefore, heavy metals may be precipitated through the adsorption of calcium carbonates, which proves that calcium carbonate precipitation has an extremely strong adsorption effect on heavy metal ions. Overall, it can be seen that the Fe and Al contents of the contaminated aged refuse and the calcium carbonate produced by the MICP process together promote the transformation of heavy metals to a more stable form after bioremediation.

### 3.8. Morphological Characteristics and Elemental Distributions of Contaminated Aged Refuse Before and After Bioremediation

**Figure 9:**
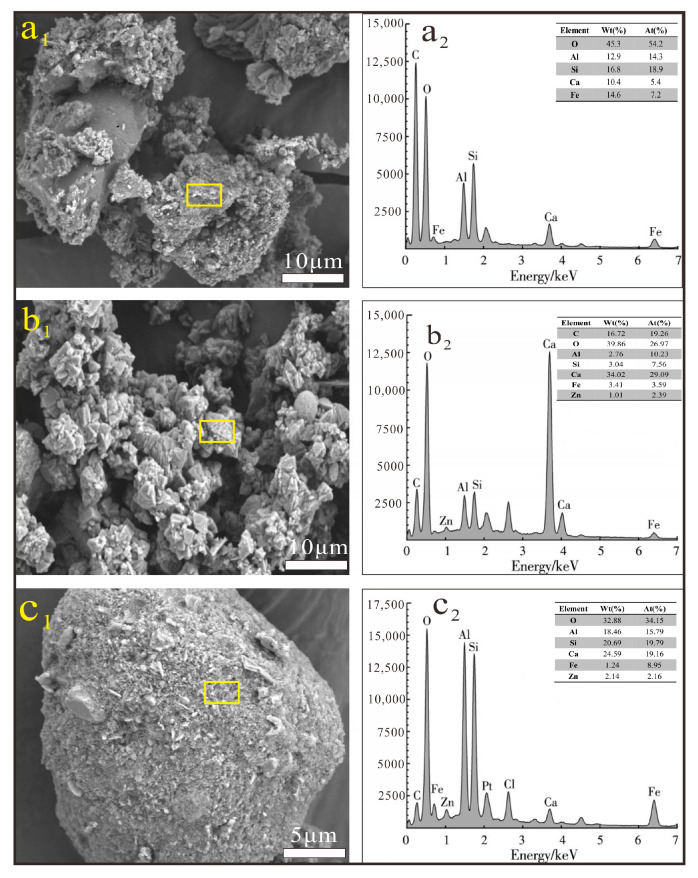
SEM-EDS results of aged refuse before and after bioremediation; (**a_1_**,**a_2_**) the results of aged refuse before bioremediation; (**b_1_**–**c_2_**) the results of aged refuse after bioremediation.

SEM-EDS was used to analyze the morphological characteristics and elemental distribution of the contaminated aged refuse before and after bioremediation, where the SEM-EDS results of the remediated samples were exemplified by the results of the Zn test group, and the other test groups had similar results. As can be seen in Figure 9a_1_, the particles of the contaminated aged refuse before bioremediation were of different sizes, with haphazard distribution and rough surfaces. This poor continuity resulted in many pores on the surface, which provided a basis for the filling of products in the MICP process. EDS results show that the elemental composition of the contaminated aged refuse before bioremediation consisted mainly of O (45.3%), Al (12.9%), Si (16.8%), Ca (10.4%), and Fe (14.6%) as seen in Figure 9a_2_. The morphological characteristics of the contaminated aged refuse are shown in Figure 9b_1_,c_1_. After bioremediation, the particle distribution was still irregular, but a large number of regular crystals were set on the surface of the samples. The calcium carbonate precipitations induced by microorganisms after MICP treatment can effectively fill the pores of the contaminated aged refuse and enhance the cementation, thus improving the compactness and strength. Therefore, the regular crystals shown in Figure 9c_1_ were calcium carbonate, and other studies have found that the restoration of dense crystals forms on the surface of the soil particles after bioremediation. The content of elements Ca (34.02%) and C (16.72%) increased after the bioremediation and contained a small amount of Zn (1.01%) (as seen in Figure 9b_2_). A related study found that bacteria acted as non-uniform nucleation sites on their surfaces, inducing the formation of calcareous shells with C, Ca, and O as major elements attached to the surface of the contaminated aged refuse. In addition, the samples contained spherical particles with elemental compositions dominated by O (32.88%), Al (18.46%), and Fe (1.24%), with small amounts of Zn (2. 14%) (Figure 9c_2_).

## 4. Discussion

### 4.1. Bioremediation Rate of Contaminated Solution and Aged Refuse

In this work, *Sporosarcina pasteurii* has the ability to remediate contaminated solution and aged refuse. However, there was a difference in the bioremediation rate. The results show that *Sporosarcina pasteurii* consistently maintained high activity, and the bioremediation rate of Cd^2+^ and Pb^2+^ in the contaminated solution increased up to 85% and 82%, respectively. In addition, *Sporosarcina pasteurii* maintained its activity and restored Zn^2+^ when Zn^2+^ concentration was in the range of 130–195 mg/L. However, as the concentration of Zn^2+^ increased from 250 to 2500 mg/L, its toxicity inhibited the expression of bacterial urease activity, resulting in a slower hydrolysis of urea until it was no longer hydrolyzed, which prevented the completion of the process of bio-precipitations. The toxicity of Cr (III) at increased concentrations likewise inhibited the expression of bacterial urease activity, resulting in a slower hydrolysis of urea until it was no longer hydrolyzed. In this study, we provided an excess of urea, and the initial concentration of Cr (III) was relatively low, and the hydrolyzed portion could be removed completely. Moreover, by comparing the results of test 1 (as seen in Figure 4) and test 2 (as seen in Figure 5), it can be seen that the contaminated aged refuse did not inhibit the microbial activity and prevent the MICP reaction from proceeding. The results show that *Sporosarcina pasteurii* consistently maintained high activity, and the bioremediation rate of Cd^2+^ and Pb^2+^ in the contaminated aged refuse increased up to 68% and 85%, respectively. In addition, as the concentration of Zn^2+^ increased from 250 to 2500 mg/L, the bioremediation rate showed a similar downward trend. The obvious differences were that the bioremediation rate for the Cr(III) test group was zero, and the mass increase in test 2 was relatively higher, mainly because the clay particles in the aged refuse had some negative charge, which can adsorb Ca^2+^ and other cations in the mixed system, providing effective nucleation sites for carbonate precipitation and promoting the MICP reaction.

### 4.2. Bioremediation Mechanism of MICP

**Figure 10:**
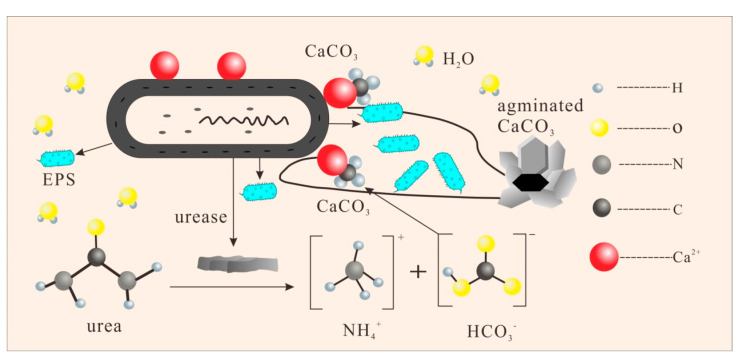
Schematic diagram of the biomineralization process.

The ability of *Sporosarcina pasteurii* to induce calcium carbonate precipitation is mainly attributed to two main features. the most important feature is its ability to secrete highly active urease, which catalyzes the decomposition of urea within the bacteria by virtue of its highly efficient urease activity that rapidly raises the concentration of carbonate and the pH value of the microenvironment around the bacteria [45,46]. Secondly, it has been shown that the surface of *Sporosarcina pasteurii* (including the cell wall and extracellular polymers) has more negative surface charges than non-mineralized bacteria and has a stronger adsorption effect on cations. Therefore, positively charged Ca^2+^ in the environment can be adsorbed on the surface of *Sporosarcina pasteurii* in large quantities. Under high concentrations of carbonate and alkaline conditions, Ca^2+^ will then settle to form CaCO_3_ crystals using the cells’ nuclei. The schematic diagram of the biomineralization process for this study is shown in Figure 10.

### 4.3. The Significance and Expectation of MICP

Certain specific microorganisms in nature utilize MICP, which produce urease enzymes that decomposed urea through metabolism, and the carbonate ions produced via the decomposition of urea combine with free metal cations in nature to produce gelling crystals, thus immobilizing heavy metals in the soil. This method can significantly reduce the concentration and bioavailability of heavy metals in the soil in a relatively short period of time, and the remediation effect is remarkable. The technology uses natural microorganisms and urea as raw materials, does not produce secondary pollution, and is environmentally friendly. In addition, MICP is highly sustainable as it utilizes natural microorganisms and chemical reactions without additional energy input.

In the future, MICP is expected to play a greater role in the field of soil heavy metal remediation. With deeper research and technological development, MICP may be combined with other remediation technologies to form a more efficient composite remediation method. Moreover, the application of MICP in contaminated sites is promising, especially where rapid and low-cost remediation is required, with significant advantages.

## 5. Conclusions

In this paper, we carried out experiments of bioremediation on heavy metal-contaminated solution and aged refuse with *Sporosarcina pasteurii*, as well as microscopic experimental studies on mineralized refuse soil before and after bioremediation. The feasibility of heavy metal bioremediation with *Sporosarcina pasteurii*, the effect of bioremediation treatment on the morphological transformation of heavy metals in aged refuse, and the mechanism of bioremediation were analyzed. The results showed that *Sporosarcina pasteurii* can maintain high activity in heavy metal-polluted solution system and aged refuse. Meanwhile, MICP has the ability to remediate Cd-, Pb-, Zn-, and Cr (III)-contaminated solutions and aged refuse with high efficiency, among which the bioremediation rate of Cd, Pb, and Cr(III) could reach 98%. After bioremediation treatment, the proportion of heavy metals in carbonate-bound form and exchangeable form in aged refuse decreased, and the content of heavy metals in residual form and iron–manganese oxide binding form increased. Therefore, the mobility and activity of heavy metals decreased, and the bioremediation achieved good results. The high Fe and Al contents in aged refuse and the effective nucleation sites provided by soil particles for the precipitation and growth of calcium carbonate facilitated the MICP reaction. Together, they promote the transformation of heavy metals in contaminated aged refuse to more stable forms. Overall, the above experimental results provide a full understanding of the bioremediation of heavy metal-contaminated solution and aged refuse, which has significant environmental benefits and economic advantages.

## Figures and Tables

**Table 1:** Working conditions of Test 1.

Number	Urea (mol/L)	CaCl_2_ (mol/L)	Cd (mg/L)	Pd (mg/L)	Zn (mg/L)	Cr(III) (mg/L)
A_1_	0.5	0.5	2.5	22.5	25	12.5
A_2_			25	225	250	125
A_3_			250	2250	2500	1250

**Table 2:** Working conditions of Test 2.

Number	Urea (mol/L)	CaCl_2_ (mol/L)	Cd (mg/L)	Pd (mg/L)	Zn (mg/L)	Cr(III) (mg/L)
B_1_	0.5	0.5	50	450	500	250
B_2_			500	4500	5000	2500
B_3_			5000	45,000	50,000	25,000

**Table 3:** Basic chemical characteristic parameters of contaminated aged refuse.

pH Value	Cd (mg/kg)	Pb (mg/kg)	Zn (mg/kg)	Cr (mg/kg)	Organic Matter (g/kg)	Cation Exchange Capacity (Cmol (+)/kg)
8.4	0.75	5.22	1230	256	126	16.8

## Data Availability

The article incorporates the original contributions of this study. For additional inquiries, please contact the corresponding author.

## Supplementary Materials

The following supporting information can be downloaded at: https://www.mdpi.com/article/10.3390/microorganisms13010064/s1, Figure S1: The location map of the landfill in Suzhou, Anhui Province, China.
