# Supplementary figures and images for: Bioremediation of Heavy Metal-Contaminated Solution and Aged Refuse by Microbially Induced Calcium Carbonate Precipitation: Further Insights into Sporosarcina pasteurii
Source: Microorganisms. 2025 Jan 2;13(1):64. doi: 10.3390/microorganisms13010064 (PMC11767937; doi:10.3390/microorganisms13010064)

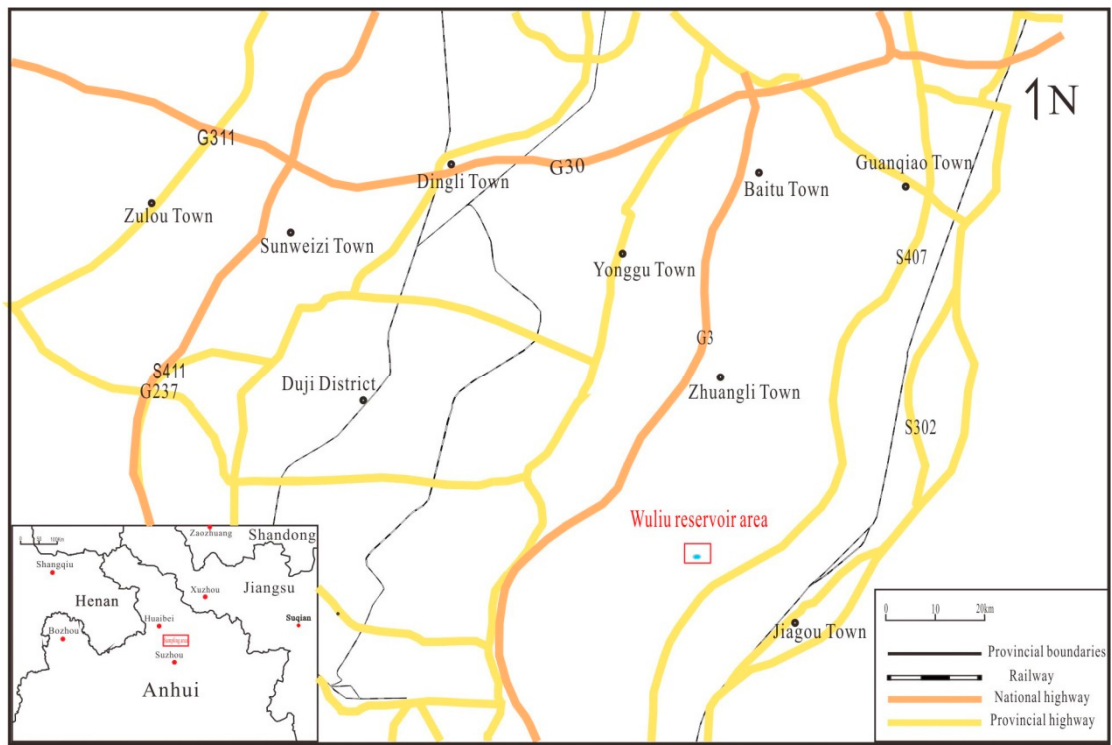

**Figure S1.** The location map of the landfill in Suzhou, Anhui Province, China.

Supplement: Supplementary file 1 [file microorganisms-13-00064-s001.zip › microorganisms-3380176-supplementary.pdf]
